# Synthesis and Evaluation of Pyridinyltriazoles as Inhibitors of p38 MAP Kinase

**Published:** 2012

**Authors:** Seyed Adel Moallem, Farzin Hadizadeh, Fatemeh Abdol Abadi, Mahmoud Shahraki, Jamal Shamsara

**Affiliations:** 1 *Biotechnology Research Centre, Mashhad University of Medical Sciences, Mashhad, Iran*; 2 *Pharmaceutical Sciences Research Centre, Mashhad University of Medical sciences Mashhad, Iran*; 3 *Medical Toxicology Research Centre, Mashhad University of Medical Sciences Mashhad, Iran*; 4 *School of Pharmacy, Mashhad University of Medical Sciences Mashhad, Iran*

**Keywords:** Inhibitors, p38 MAP kinase, Pyridinylimidazoles

## Abstract

**Objective(s):**Inhibitors of p38 MAP kinase are considered as suitable target in the treatment of inflammatory diseases such as rheumatoid arthritis and bowel inflammatory diseases. The development of 5-alkylthio-1-aryl-2-(4-pyridinyl) triazoles as inhibitors of p38 MAP kinase is described. These are analogues of 4- pyridinyl imidazole p38 MAP kinase inhibitor reported by Merck Research Laboratories, in which imidazole ring has been replaced with triazole.

**Materials and Methods:**Reaction of pyridine-4-carboxylic acid hydrazide 1 and arylisothiocyanate (2a, b) gave the intermediate thiourea derivative 3a, b (Figure 2). Refluxing of the latter in aqueous saturated sodium carbonate gave 1-aryl-5-mercapto-2-(4-pyridinyl) triazoles 4a, b. Treatment of 4a, b with alkyl iodide afforded the desired 5-alkylthio-1-aryl-2-(4-pyridinyl) triazoles (5a-d). P38 MAP kinase inhibitory activity of the synthesized compounds was evaluated *in vitro* by ELISA method and also by molecular docking.

**Results:**Compound 5c at 1 µM concentration and compound 5d at 1 µM and 10 µM significantly inhibited the p38 phosphorylation. These inhibitory effects are equal to those of standard compound SB202190 and no significant differences were observed.

**Conclusion:**We demonstrated that both tested compounds have inhibitory effect on p38 MAP kinase and we did not find significant difference between their inhibitory effects and those of standard inhibitor SB202190.

## Introduction

**Figure 1:**
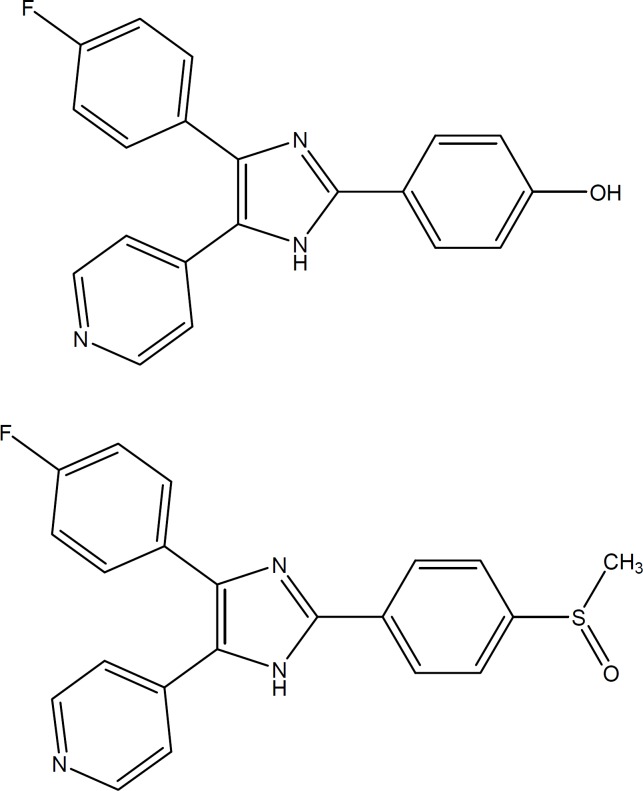
(a) SB 202190, (b) SB 203580

Rheumatoid arthritis and other chronic inflammatory diseases constitute a major therapeutic challenge, usually not sufficiently met by the classical anti-inflammatory medications. Recent research efforts provided new insights into the molecular basis of these pathologies and disclosed new opportunities for developing improved drugs directed to the chemical mediators of the disease. The enzyme p38 MAP kinase plays a central role in the signal transduction cascade that leads to the production of both the proinflammatory cytokines, TNF-α and IL-1β. Thus representing an attractive therapeutic target for novel anti-inflammatory therapies (1-4). A number of p38 inhibitors belonging to different structural families have been developed as potential anti-inflammatory drugs, and some of them progressed into clinical trials. Many of the published p38a inhibitors possess a vicinal aryl/4- pyridinyl-heterocycle arrangement exemplified by the pyridinylimidazole SB-202190 (Figure 1) (5). It was suggested that the p38 MAP kinase inhibitors could also be new agents for treatment of Alzheimer’s disease (6). 

These compounds act as competitive inhibitors at the ATP binding site of the p38 MAP kinase. Crystal structures of p38-inhibitor complex have shown that this class of compounds utilizes two key binding interactions. The pyridine nitrogen forms a hydrogen bond with the main chain N–H of Met 109 and the aryl substituent penetrates into a hydrophobic pocket not accessed by ATP. Conversely, variations in the 2-substituent of SB 203580 showed that this moiety does not define a key structural feature for p38 inhibitors (5,7-9). 

**Figure 2:**
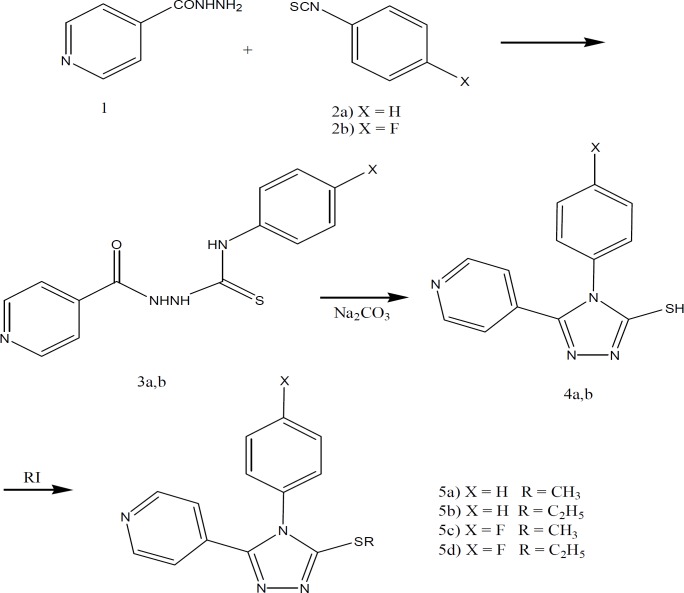
Synthesis of 5-alkylthio-1-aryl-2-(4-pyridinyl) triazoles (5a-d)

In this study we described synthesis and p38 MAP Kinase inhibitory activities of 5-alkylthio-1-aryl-2-(4-pyridinyl) triazoles (5a-d) in which imidazole heterocycle had been replaced with triazole ring (Figure    1 ). Reaction of pyridine-4-carboxylic acid hydrazide 1 and arylisothiocyanate (2a, b) gave the intermediate thiourea derivative 3a, b** (**Figure 2**)**. Refluxing of the latter in aqueous saturated sodium carbonate overnight gave 1-aryl-5-mercapto-2-(4-pyridinyl)triazoles 4a, b. Treatment of 4a, b with the corresponding alkyl iodide afforded the desired 5-alkylthio-1-aryl-2-(4-pyridinyl)triazoles (5a-d).

## Materials and Methods

***Chemical synthesis***

Melting points were determined on Electrothermal Capillary apparatus. ^1^H NMR were obtained on Bruker Ac-80 spectrophotometer and chemical shifts () were in ppm relative to internal tetramethylsilane. Errors of elemental analyses were within 0.4% of theoretical values.

***General procedure for preparation of 1-(4-pyridinyl carbonyl)-2-(arylaminothiocarbonyl) hydrazine 3a, b***

To a stirring solution of pyridine-4-carboxylic acid hydrazide (1.5 mmoles, 205.71 mg) in ethanol (5 ml) was added dropwise arylisothiocyanate (1.5 mmoles). The resulting mixture was stirred for 24 hr. The precipitate was filtered and recrystilized from ethanol to give 3a, b.

*1-(4-Pyridinyl* *‌* *carbonyl)-2-(phenylaminothiocarbonyl) hydrazine* (3a) 

This compound was obtained in 84% yield; M.P. 186-190 ^o^C; ^1^H NMR (DMSO-d_6_):. 9.8 (bs, 1H, NH), 8.8 (bs, 2H, NH), 7.8(d, 2H, H-pyridine), 6.9-6.2 (m, 7H, aromatic). *Anal*. Calcd. For C_13_H_12_N_4_OS: C, 57.34; H, 4.44; N, 20.57. Found: C, 57.43; H, 4.33; N, 20.64.

*1-(4-Pyridinyl* *‌* *carbonyl)-2-(4* *‌* *-* *‌* *fluorophenylaminothiocarbonyl)* *‌hydrazine *(3b) 

This compound was obtained in 89% yield; M.P. 197-203 ^o^C; ^1^H NMR (DMSO-d_6_): . 9.8 (bs, 1H, NH), 8.8 (bs, 2H, NH), 7.8(d, 2H, H-pyridine), 6.9-6.2 (m, 6H, aromatic). *Anal*. Calcd. For C_13_H_11_FN_4_OS: C, 53.78; H, 3.82; N, 19.30. Found: C, 53.81; H, 3.73; N, 19.38.

***General procedure for preparation of 1-aryl -5-mercapto -2-(4-pyridinyl) triazoles (4a, b)***

To a stirring solution of 3a, b (1.5 mmoles), a saturated solution of sodium carbonate was added and the resulting mixture was refluxed overnight. After cooling, the mixture was acidified by adding conc. HCl. The precipitate was filtered and recrystalized from methanol to give 4a, b. 

*1-Phenyl-5-mercapto-2-(4-pyridinyl)triazoles* (4a)

This compound was obtained in 95% yield; M.P. 280-285^o^C; ^1^H NMR (DMSO-d_6_) : .7.6 (d, 2H, H-pyridine), 6.3 (m, 7H, aromatic). *Anal*. Calcd. for C_13_H_10_N_4_S: C, 61.40; H, 3.96; N, 22.03. Found: C, 61.34; H, 3.83; N, 22.12.

*1-(4-Fluorophenyl)-5-mercapto* *‌* *-2-(4-pyridinyl) triazoles* (4b)

This compound was obtained in 90% yield; M.P. 272-275 ^o^C; ^1^H NMR (DMSO-d_6_): .7.9 (d, 2H, H-pyridine), 6.3 (m, 6H, aromatic). *Anal*. Calcd. for C_13_H_9_FN_4_S: C, 57.34; H, 3.33; N, 20.58. Found: C, 57.23; H, 3.39; N, 20.46.

***General procedure for preparation of 1-aryl -5-alkylthio -2-(4-pyridinyl) triazoles (5a-d)***

To a stirring solution of 4a, b (14.5 mmoles) in methanol (200 ml) , 1N sodium hydroxide was added until we had a clear solution (about 20 ml). Then, alkyl iodide (15.3 mmole) was added slowly. The resulting mixture was stirred overnight. The methanol was evaporated in vacuum and 150 ml of water was added to the residue. The aqueous layer was extracted with chloroform (3 x 60 ml). The organic was evaporated in vacuum to give 5a-d.

*1-Phenyl -5-methylthio -2-(4-pyridinyl) triazoles (5a)*

This compound was obtained in 85% yield; M.P. 163-166^o^C; ^1^H NMR (CDCl_3_): .8.5 (d, 2H, H-pyridine), 7.5 (m, 7H, aromatic), 7.5 (m, 7H, arom), 2.7 (s, 3H, CH_3_). *Anal*. Calcd. for C_14_H_12_N_4_S: C, 62.66; H, 4.51; N, 20.88. Found: C, 62.53; H, 4.63; N, 20.71.

*1-Phenyl -5-ethylthio -2-(4-pyridinyl)triazoles* (5b)

This compound was obtained in 90% yield; M.P. 177-180^o^C; ^1^H NMR (CDCl_3_): .8.6 (d, 2H, H-pyridine), 7.2-7.7 (m, 7H, aromatic), 3.4 (q, 2H, CH_2_), 1.5 (t, 3H, CH_3_). *Anal*. Calcd. for C_15_H_14_N_4_S: C, 63.80; H, 5.00; N, 19.84. Found: C, 63.89; H, 5.25; N, 19.73.

*1-(4-Fluorophenyl)-5-methylthio* *‌* *-2-(4-pyridinyl)triazoles* (5c)

This compound was obtained in 85% yield; M.P. 183-185 ^o^C; ^1^H NMR (CDCl_3_): .8.5 (d, 2H, H-pyridine), 7.5 (m, 7H, aromatic), 7.5 (m, 6H, arom), 2.7 (s, 3H, CH_3_). *Anal*. Calcd. for C_14_H_11_FN_4_S: C, 58.73; H, 3.87; 19.57. Found: C, 58.84; H, 3.73; N, 19.62.

*1-(4-Fluorophenyl)-5-ethylthio* *‌* *-2-(4-pyridinyl) triazoles *(5d)

This compound was obtained in 87% yield; M.P. 137-140^o^C; ^1^H NMR (CDCl_3_): .8.6 (d, 2H, H-pyridine), 7.2 (m, 6H, aromatic), 3.4 (q, 2H, CH_2_), 1.6 (t, 3H, CH_3_). *Anal*. Calcd. for C_15_H_13_FN_4_S: C, 59.98; H, 4.36; N, 18.65. Found: C, 60.01; H, 4.49; N, 18.71.

***MAP kinase inhibition assay***

We used CASE^TM^ (cellular activation of signaling ELISAs) kit with Cat. No. FE-003 (SABiosciences, USA) to determine the possible inhibitory effects of our synthesized compounds on p38 MAP Kinase. All compounds were dissolved in DMSO / water mixture max. 0.03%. The kit is a cell based ELISA that determines level of total and activated form of p38 at a same time on cells fixed in a 96-well culture plate. This colorimetric assay compares relative amount of phosphorylated p38 to total p38 protein among the samples to identify the effects of treatment. Each assay was done following the manufacturer’s instruction.

***Molecular modeling*** ***and docking procedure***

Molecular geometry of the synthetic compounds was optimized in Molecular Operating Environment (MOE) software by using of Force Field-MMFF94x.

The crystal structure of human p38 MAP kinase complexed with SB203580 (3GCP) (Figure 1) was downloaded from the Protein data Bank Brookhaven (www.rcsb.org) and opened with MOE software. The complexed inhibitor was removed when using the synthesized compounds as ligands for docking. MOE was also used to calculate binding energy of the best intermolecular interaction between the ligand and the enzyme binding site as given in the software manual. MOE–Dock is used to search for favorable binding conﬁgurations between the ligand and the macromolecule.

Our two compounds for docking were drawn in mol format and all hydrogens were added. They were energy minimized using Hamiltonian-Force Field-MMFF94x and the partial charges were also calculated.

Docking procedure of p38 MAP kinase was as following: (a) the co-crystallized SB203580 was identiﬁed; therefore, the binding site was identiﬁed with its residues (2). Ligand interactions were computed for the X-ray co- crystallized SB203580 to reveal the different types of interaction as a validation for the coming docking procedure (3). The co-crystallized SB203580 is then removed and the selected synthesized compounds are used instead (4). The docking was done with the default setting of the MOE–DOCK as following: a. the option: rotated bonds were selected to give ﬂexible ligand-rigid receptor docking; b. the scoring function was London dG with a replacement of Alpha Triangle; c. 30 conformers of the ligand were retained with highest and best score by default; d. the top score ligand-receptor docking was then demonstrated by 2D ligand-receptor interactions.

## Results

**Figure 3:**
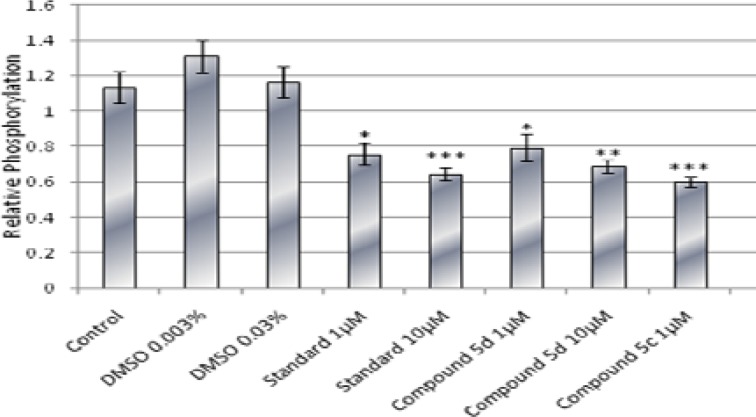
Effect of compounds 5c, 5d at 1 and 10 µM, DMSO at 0.03% and 0.003 %, and standard (SB202190 at 1 and 10 µM) on relative phosphorylation of p38. (Each data was obtained from 8 well measurements and reported as mean±SEM), *:*P*< 0.05 **:*P*< 0.01 ***: *P*< 0.001

DMSO was used as a solvent in our assay. It did not have any significant effect on p38 activity or cell viability in maximum used concentration (0.03%) compared to control. Compound 5c at 1 µM concentration and compound 5d at 1µM and 10 µM significantly inhibited the p38 phosphorylation. These inhibitory effects were equal to those of standard (compound SB202190) and no significant differences were observed (Figure 3). However, to assess the specificity of these compounds we need to determine the possible inhibitory effects of them on other pathways such as ERK, JNK.

**Figure 4:**
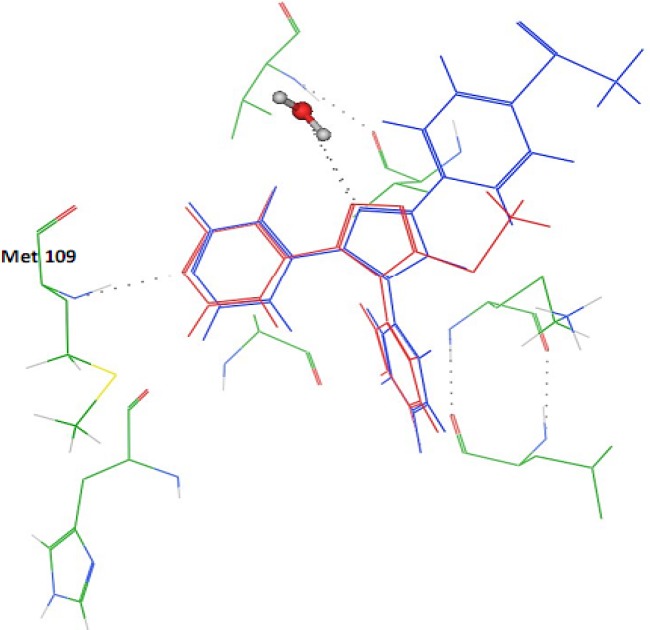
Relative binding orientations of SB 203580 (blue) and compound **5c** (red)

**Figure 5:**
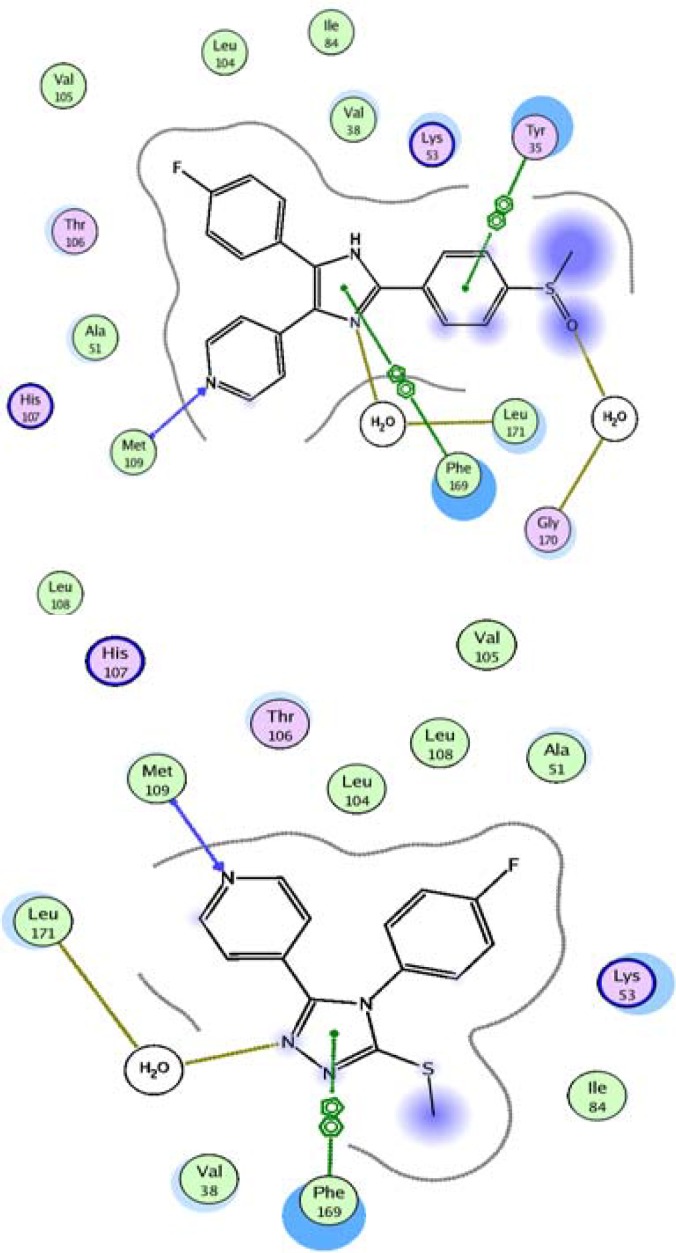
Docking of p38 MAP kinase with (a) SB 203580 and (b) compound 5c in 2D diagram (a) in the binding pocket of p38 MAP kinase

*In silico* docking experiment showed that in the complex of SB 203580 and p38 MAP kinase (PDB ID: 3GCP), the 4-fluorophenyl moiety of the inhibitor binds to hydrophobic pocket, where Thr 106 acts as a gatekeeper. In several related kinases, Thr 106 is replaced by amino acids with larger side chains (such as Met) which explains the selectivity of inhibitors found in the class of 4-aryl- 5-(pyridin-4-yl) imidazoles and the pyridin-4-yl moiety is important for inhibitory potency and generates a crucial hydrogen bond with the backbone amino group of Met 109 (Figure 4 and 5a) (10). It was also found that Ph169 has a charge transfer interaction with imidazole nucleus in SB 203580 (Figure 5a). In docking study on our synthesized compound 5c, we observed similar interactions with p38 MAP kinase (Figure 4 and 5b).

## Discussion

Pyridinyl imidazoles such as the prototype SB 203580 are known for potent inhibition of p38 MAP kinase. Inhibitors of this class compete with the original co-substrate ATP for binding to the ATP binding pocket of the kinase. Unlike the native p38 MAP kinase co- substrate ATP, pyridinyl imidazole inhibitors bind to the active site of both the active (bis-phosphorylated) and inactive forms of the kinase. The essential pharmacophore is the 4-aryl-5-(pyridin-4-yl) imidazole. 

In docking study, both SB 203580 and our synthesized compounds 4-fluorophenyl and pyridin-4-yl moieties occupied the same positions. There was also found a hydrophobic pocket for methylthio (SMe) residue between Phe169 and Ile84. It was also previously shown in SAR of these inhibitors that polar substitution at para position of the phenyl ring leads to enhance binding (11). In addition, the triazole ring of the synthesized compounds had charge transfer interaction with Phe169, the same as what was seen in SB 203580 (Figure 5b). In accordance to docking results, the inhibitory effects of 5c and 5d were equal to those of standard compound SB202190 and no significant differences were observed.

## Conclusions

In this study, we presented the synthesis of a series of novel 5-alkylthio-1-aryl-2-(4-pyridinyl) triazoles as analogues of pyridinylimidazole p38 MAP kinase inhibitors in which imidazole heterocycle has been replaced with triazole ring and evaluation of their inhibitory potency against p38 MAP kinase. Both of our synthesized compounds have a significant inhibitory effect on phosphorylation caused by p38 MAP kinase.
